# Perception among medical students in Riyadh, Saudi Arabia, regarding alcohol and substance abuse in the community: a cross-sectional survey

**DOI:** 10.1186/1747-597X-5-2

**Published:** 2010-01-22

**Authors:** Ali I Al-Haqwi

**Affiliations:** 1 Family and Community medicine, King Saud Ben Abdul-Aziz University for Health Sciences (KSAU-HS), Riyadh, Saudi Arabia

## Abstract

**Background:**

This study was conducted to examine the perception and views of medical students regarding the extent of alcohol and substance abuse in the community and the possible predisposing factors for this problem.

**Methods:**

It is a cross-sectional study involving samples from two medical colleges in Riyadh, Saudi Arabia. The students who decided to participate in the study without the offer of any incentives filled an anonymous, self administered questionnaire which had been designed to meet the purpose of the study.

**Results:**

Two hundred and fifteen out of three hundred and thirty students (65% response rate) participated in this study. About 75% of them believe that alcohol and substance abuse is a common problem in the community. Students' views also correspond with the reported view that the problem is mainly present in young adult males. Married males and senior students perceived the problem as more serious than their other colleagues. Students perceived that alcohol was the most commonly abused drug in the community, followed by amphetamines, heroin, cannabis and cocaine. They believe that influence of friends, life stressors, tobacco smoking and curiosity are the most important predisposing factors for abuse of alcohol and other substances. According to the students' perception, the main beneficial effect of alcohol and substance abuse was stress alleviation. About 3% of the students have also indicated that they may use alcohol or some other substance in the future.

**Conclusion:**

Despite scarce information on the subject and a strong religious belief in Saudi Arabia against the use of alcohol and other addictive substances, a significant majority of the medical students in Riyadh, the capital of Saudi Arabia, perceived that alcohol and substance abuse is a common problem in the community. Some students appear to perceive the seriousness of the problem less than others. Efforts are needed to educate young men and women at an early stage of their academic life, as a medical student about the existence of this problem in the community, its consequences and predisposing factors. Teaching teenagers and young adults about stress coping strategies may be of special importance in reducing the risk of alcohol and substance abuse.

## Background

Abuse of alcohol and other substances is becoming a considerable health and social problem in the world [1]. More than seventy nine thousands deaths are attributed to alcohol in the United States of America (USA) annually, which makes it one of the leading causes of death in that country [2]. In the USA, about 8.3% of persons above the age of 12 have indicated using at least an illicit drug in the past month [3].

Moreover, in the USA, substance abuse disorders are expected to increase significantly by 2020 [4].

This problem rises significantly in college students, as about 23% of them were found to use substances meeting the criteria of substance abuse and dependence [5].

Studies in Turkey and other parts of the world have shown that excessive alcohol consumption is common among medical students and physicians [6-8]. They are at risk of developing serious adverse effects because of their usage [8], which necessitates an urgent call for implementing preventive and counseling programs to reduce alcohol-related harm among college students and other young adults [9].

Despite limited information, alcohol and substance abuse has been confirmed in mainly Muslim countries including Gulf Arab nationals [10]. Most commonly abused drugs in these countries are alcohol, heroin and hashish. Available literature demonstrated that substance abuse behavior is even more prevalent among Arab adolescents compared to that of Jewish [11,12].

Consumption of alcohol or other addictive substances is considered as "illegal" in Saudi Arabia, as the law is based on Islamic Sharia rules, which forbid consumption of any amount of alcohol and other forms of substances. Therefore, for religious and legal reasons, any use of alcohol is considered as abuse by Saudi general public.

Information about the extent of this problem in Saudi Arabia is limited. However, statistics of the Ministry of Health in Saudi Arabia showed a substantial increase in specialized psychiatric hospitals outpatient visits and inpatient admissions in 2007 compared to 2003 [13]. Some of this increase could be due to problems related to alcohol and substance abuse. In absence of official reports about the magnitude of substance abuse, this could be used as an indirect indicator of the increased demand on such services.

Moreover, reports of trafficking of illegal drugs, despite the threats of severe punishment reflect the seriousness of this issue in the country [14].

Attitudes and practices of physicians and future physicians towards alcohol and substance abuse is a topic of significant importance and deserved to be examined. Their views will determine the extent of their involvement in the management programs for this serious problem, detrimental to the health of the community.

This study aimed to examine the views and perceptions of medical students about the extent of substance abuse including alcohol in the community and the predisposing factors related to this problem.

## Methods

This is a cross-sectional study. This study was carried out during the month of June 2009 and involved students from two medical colleges in Riyadh, the capital of Saudi Arabia. These two colleges were selected in order to have a homogenous sample of students as they were the only colleges adopting "Problem Base Learning" curriculum at the time of the study.

All the students of these two colleges were invited to participate in the study by filling an anonymous self-administered questionnaire, which was prepared to meet the objectives of this study. The questionnaire included demographic characteristics of students including their study level and GPA. Study level stands for the academic stage of the students which ranges from level1, junior to level 5, senior level. GPA stands for Grade Point Average, which reflects the academic performance of students.

The questionnaire included as well students' opinion if they perceived that substance abuse and alcohol use is a common problem in the community?, their views of commonly abused drugs, the predisposing factors and the beneficial effects of substances if any, and whether they think that they may use alcohol or other substances in the future. The questionnaire was constructed utilizing some available published data [15-17], which was further piloted among 10 students to determine clarity of questions to students. Some questions were modified according to students' comments.

The questionnaire was mailed to all students of the two medical colleges. Personal information that could reveal students' identity including name and number was not required. This is to ensure confidentiality and to encourage more participation and expression of opinions.

Students were instructed to return the filled questionnaire to the author through the post mail.

Data was coded, entered and analyzed using the Statistical Package for Social Sciences (SPSS), version (17). A descriptive analysis was done to summarize information. This was done by calculating the number and percent for categorical variables, whereas the mean and standard deviation (SD) was calculated for continuous variables. Chi-square test was used to test significance between variables. Moreover, Jonckheere-Terpstra test was used to assess association between categorical variables and the ordinal variables for the opinion. Similarly, the Spearman correlation was used to assess the association between the continuous variables and the ordinal variables for the opinion. A p-value less than 0.05 was considered statistically significant.

The proposal of this study was reviewed and approved by the research ethics committee at the King Abdullah International Research Center.

## Results

A total number of 215 out of 330 students participated in this study, which makes a response rate of 65%. The mean age of the participants was 21 years (+/- 3 SD). Male students constituted 77% and more than 90% were single. Other characteristics of the study sample are shown in Table 1.

**Table 1:** Characteristics of the participants.

	Number	Percentage %
Sex		
• Male	165	77
• female	50	23

Marital status		
• Single	200	93
• Married	15	7

Study level		
• 1	86	40
• 2	35	17
• 3	27	13
• 4	46	22
• 5	17	8

GPA		
• Excellent	49	23
• Very good	78	36
• Good	54	25
• Fair	16	7

Table 2 shows that more than 75% of the participants perceived that alcohol use and substance abuse is a real problem in Saudi community and about 15% could not determine if this problem existed or not. The majority of the participants believed that substance abuse is a problem of young males of 15-30 years age group.

**Table 2:** Students awareness of substance abuse as a problem and some of its features.

	Frequency	Percent (%)
**Substance abuse is a common problem in Saudi community**		
• Strongly Agree	69	32%
• Agree	95	44%
• Disagree	7	3.2%
• Strongly Disagree	4	2%
• Don't know	31	14.4%
• Unavailable Data	9	5%

**Substance abuse affect mainly the following age group:**		
• <15	19	9%
• 15-30	167	77.3%
• 30-45	4	2%
• >45	1	1%
• Unavailable Data	24	11%

**Substance abuse affect mainly:**		
• Males	179	83%
• Females	3	1.4%
• Don't know	24	11.1%
• Unavailable data	9	5%

The effect of students' characteristics in their opinions of the magnitude of the problem is shown in Table 3. Males and married students think that the problem of substance abuse is more common than their other colleagues (Jonckheere-Terpstra z = 3.28, p < 0.001 and z = 3.97, p < 0.0001, respectively). Perceived magnitude of a substance abuse problem was also significantly correlated with study year (Spearman r = 0.46, p < 0.0001) and GPA (Spearman r = 0.15, p < 0.05).

**Table 3:** Students perception of substance abuse as a problem and students characteristics.

	Opinion										
	**Strongly agree**		**Agree**		**Disagree**		**Strongly disagree**		**Don't know ¥**		**Total**
	**N**	**%**	**N**	**%**	**N**	**%**	**N**	**%**	**N**	**%**	

Sex**§**											
• Male	58	36	79	50	2	1.25	1	0.6	20	12.5	160 *
• Female	11	24	16	34	5	11	3	6.5	11	24	46
											206

Marital Status**§**											
• Single	57	30	93	49	6	3	4	2	31	16	191
• Married	12	86	2	14	0	0	0	0	0	0	14 *
											205

Study level¶											
• 1	17	21	29	36	6	7.5	4	5	24	30	80
• 2	4	12	24	70	1	3	0	0	5	15	34
• 3	15	54	13	46	0	0	0	0	0	0	28
• 4	21	75	23	82	0	0	0	0	1	2	45 *
• 5	12	75	4	25	0	0	0	0	0	0	16 *
											203
GPA¶											
• Excellent	10	22	20	43	2	4	1	2	13	28	46
• Very good	24	31	41	53	2	3	2	3	8	10	77
• Good	24	46	23	44	2	4	1	2	2	4	52
• Fair	4	27	4	27	1	6	0	0	6	40	15
											190

*P-value statistically significant

¥: This response was taken as "neither agree nor disagree"

§: Jonckheere-Terpstra test was used to assess significance

¶: Spearman correlation was used to assess significance

Majority of students perceive that alcohol is the most commonly drug of abuse in the community, followed by amphetamines, heroin, cannabis and cocaine.

They believe that influence of friends is the most important predisposing factor leading to abuse of alcohol and other substances, followed by life stresses, tobacco smoking and the curiosity related to experimentation. The factors that had the least contribution to substance abuse, in their views were; physical diseases and inadequate recreational facilities, as shown in Table 4.

**Table 4:** Perceived risk and protective factors for substance use.

Factors	Frequency	Percent (%)
Friends	171	79.2%

Life Stress	129	60%

Tobacco Smoke	125	58%

Curiosity/For experimentation	124	57.4%

Living away from home	119	55.1%

Psychological diseases	117	54.2%

Social reasons	115	53.2%

Family Members	109	51%

Study stress including exams	101	47%

Lack of knowledge of harmful effects	100	46.3%

Religious/Moral unacceptability	87	40.3%

Inadequate recreational facilities	66	31%

Physical diseases	50	23.1%

About a third of the participants indicated that abuse of alcohol and other substances could have some beneficial effect in the form of alleviation of stress. However, students generally believe that overall there are very little benefits, if any with substance abuse.

About 70% of the participants indicated that they don't intend to use alcohol or other substances in the future, and about 27% are not sure about their future intentions. About 3% of the students indicated that they might use alcohol or other substances in the future.

## Discussion

The religious and legal rules in Saudi Arabia forbid consumption of any amount of alcohol and other forms of substances.

Despite this fact, about three quarter of medical students in this study have perception that alcohol and substance abuse is a real problem in the community. This view could be based on the encountered experiences of substance related problems of abuse in the community which may be a result of recent socioeconomic and rapid lifestyle changes, especially in the younger section of the Saudi population. However, lack of enough dissemination of information on this issue might explain that a considerable percentage of medical students could not indicate whether alcohol and substance abuse is a common problem in the community or not?

Most of the medical students have indicated that the substance abuse is a behavior usually practiced by young adult males. This is in-keeping with the available descriptive statistics from studies of substance abuse behavior in Saudi Arabia [18,19].

The view of students about presence of substance abuse in the community was shown to be highly affected by their gender, marital status and level of study i.e.: being a married male at a senior level of study. This probably raises the need of educating other subgroups of students, especially junior and female students to gain more insight into the problem at an earlier stage of their career.

Alcohol, amphetamines, heroin and cannabis were thought to be the most frequently abused substances in the community. This view corresponds with that of primary care physicians in Saudi Arabia in considering alcohol as the most commonly abused substance [15]. It has also been reported that Alcohol abuse is the main substance abuse forms in addiction centers and specialized psychiatric centers in Saudi Arabia [18]. Moreover, it has been noticed that the trend of abusing amphetamines and cannabis is on the rise [19].

Friends and peer pressure, life stresses, tobacco smoking and curiosity were felt to be the main factors that may predispose to alcohol and other substance abuse. Peer pressure and curiosity were also found to be the main reasons of initiating drug use in a published study [20]. This initiation and experimentation of substance as a result of curiosity and peer influence might lead to abuse and dependence later in life. One twin study points to the contribution of environmental factors on the transition from early alcohol use to alcohol or other substance abuse and dependence [21].

General life stresses and those especially associated with the study of Medicine should be treated carefully as they could affect the general well-being of medical students and their academic performance and may lead to some form of alcohol or substance abuse [13,22,23].

Interestingly, more than a third of students indicated that alcohol and other substances could have a beneficial effect as stress alleviation, which is similar to that found in a survey of Pakistani medical students [16]. This is of significant importance as students should be taught safe and more effective coping strategies for the stressors in their lives. This is particularly true for high risk students and those who have indicated that they may use alcohol and other substance in future.

## Conclusion

Despite scarce information on the subject and a strong religious belief in Saudi Arabia against the use of alcohol and other addictive substances, medical students think that alcohol and substance abuse is a common problem in the community. The awareness of some students appears to be less than others. Efforts are needed to educate medical students at an early stage of their study about the problems, its consequences and predisposing factors. Teaching and training students stress coping strategies may be of special importance to reduce risk of alcohol and substance abuse.

## Recommendations

Based on the findings of this study, the following recommendations are suggested to policy makers:

1. Awareness programs for medical college students and other youth groups about the hazards of alcohol and other substances abuse are badly needed.

2. Special emphasis should be made for teaching certain issues such as the role of tobacco smoking, peer influence and life stressors as predisposing factors for the alcohol and substance abuse problems.

3. Counseling programs should be implemented to support students, especially during difficult periods of their study of medicine.

4. Multiple and regular "stress coping strategies" sessions could be organized for medical students to help them to cope with life stressors and to minimize the possibility of use of alcohol or other substances.

## Competing interests

The author declares that they have no competing interests.
